# Switching the Sphingolipid Rheostat in the Treatment of Diabetes and Cancer Comorbidity from a Problem to an Advantage

**DOI:** 10.1155/2015/165105

**Published:** 2015-03-19

**Authors:** Nikolas K. Haass, Najah Nassif, Eileen M. McGowan

**Affiliations:** ^1^The University of Queensland, The University of Queensland Diamantina Institute, Translational Research Institute, Brisbane, QLD, Australia; ^2^The Centenary Institute, Newtown, NSW, Australia; ^3^Discipline of Dermatology, University of Sydney, Camperdown, NSW, Australia; ^4^School of Medical and Molecular Biosciences, University of Technology Sydney, Ultimo, NSW 2007, Australia; ^5^Sydney Medical School, University of Sydney, Camperdown, NSW 2050, Australia

## Abstract

Cancer and diabetes are among the most common diseases in western societies. Epidemiological studies have shown that diabetic patients have a significantly higher risk of developing a number of different types of cancers and that individuals with comorbidity (cancer and diabetes/prediabetes) have a poorer prognosis relative to nondiabetic cancer patients. The increasing frequency of comorbidity of cancer and diabetes mellitus, mainly type 2 diabetes, has driven the development of therapeutic interventions that target both disease states. There is strong evidence to suggest that balancing the sphingolipid rheostat, ceramide—sphingosine—sphingosine-1-phosphate (S1P) is crucial in the prevention of diabetes and cancer and sphingosine kinase/S1P modulators are currently under development for the treatment of cancer and diabetes. This paper will highlight some of the complexities inherent in the use of the emerging sphingosine kinase/S1P modulators in the treatment of comorbidity of diabetes and cancer.

## 1. Introduction

Cancer and diabetes mellitus are two of the most prevalent diseases worldwide. An estimated 347 million people worldwide suffer from diabetes [1]. The World Health Organization (WHO) projects this disease to become the 7th leading cause of death by 2030 [2]. Cancer is the 2nd most prevalent disease worldwide [3, 4]. Whilst there is an increasing awareness of a strong association between the two diseases, both for cancer incidence and prognosis, the biologic links between diabetes and cancer risk are not well defined [5–7]. Type 2 diabetic patients have a greater propensity to develop cancer, and cancer and diabetes share many risk factors [8]. Some epidemiological studies suggest increased mortality in cancer patients with preexisting diabetes [9]. With the increasing likelihood of comorbidity of cancer and diabetes and the potential of increased mortality in these patients [9–11], understanding the aetiology underlying both diseases will aid in the development of more efficacious treatments.

Sphingosine kinase (SphK) is an important signalling enzyme that catalyses the phosphorylation of the lipid sphingosine to form sphingosine-1-phosphate (S1P) and has been implicated in the pathology of both diabetes and cancer [7, 12–17]. SphK plays a critical role in balancing the relative levels of the two signalling molecules controlling cellular metabolic processes such as cell proliferation, survival, apoptosis, adhesion, and migration [18–20]. Hence there is a strong motivation for the development of SphK/S1P modulators for therapeutic interventions to target patients with comorbidity of diabetes and cancer. This paper, as part of the special issue on “Hijacking the metabolic regulation in cancer and diabetes,” aims to highlight the complications arising from targeting the SphK1/S1P rheostat, by the S1P modulators, for cancer therapy in patients with prediabetes/diabetes.

## 2. Type 1 and Type 2 Diabetes 

Type 1 and type 2 diabetes are complex diseases characterised by progressive failure of the insulin producing pancreatic *β*-cells [21]. The mechanisms of pancreatic *β*-cell death in type 1 and type 2 diabetes have very few similarities [22]. Type 1 diabetes is caused by an autoimmune attack resulting in the loss of the insulin producing *β*-cells and loss of insulin secretion whereas type 2 diabetes is characterised by insulin resistance, which can lead to a relative state of hyperinsulinaemia (overproduction of insulin) to maintain normal glycaemia and eventually results in *β*-cell failure. Approximately 10% of diabetic patients have type 1 diabetes (usually starting in childhood or younger age), and these patients have an absolute requirement for insulin therapy requiring daily dosage of insulin. Type 2 diabetes is the most common, making up approximately 90% of all cases. In most instances these patients are noninsulin dependent; however, over time, they may require insulin to maintain glycaemic control. The onset of type 2 diabetes is usually later in life and is associated with obesity and a sedentary lifestyle. Saturated fatty acids associated with obesity, such as palmitate, are lipotoxic towards the pancreatic *β*-cells, exerting a double hit: insulin resistance and reduced pancreatic *β*-cell survival [23, 24]. Skeletal muscle also plays a major role in the pathology of insulin resistance as this tissue is important for whole body insulin-stimulated glucose removal [25]. Thus perturbation of insulin signalling in skeletal muscle is a key factor in type 2 diabetes development. Complications of both type 1 and type 2 diabetes include cardiovascular disease, neuropathy, retinopathy, and kidney failure [21].

## 3. Obesity, Diabetes, and Cancer

**Figure 1:**
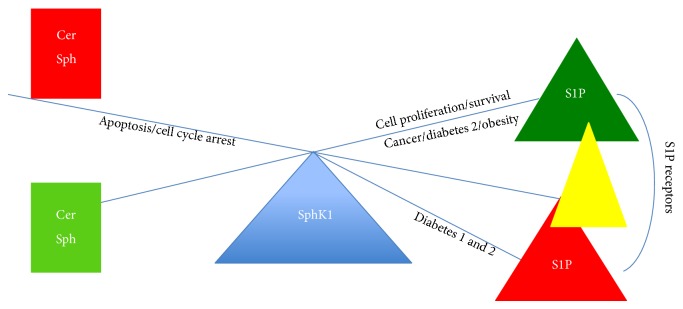
The swinging pendulum. Overexpression of SphK1 activates S1P and favours cell proliferation and survival. S1P overexpression is associated with cancer progression, type 2 diabetes complications such as inflammation, metabolic dysfunction, cardiovascular problems, nephropathy, retinopathy, and neuropathy. Loss of S1P can affect pancreatic *β*-cell proliferation and is associated with the progression of both type 1 and type 2 diabetes. Therapeutic intervention involving binding to specific S1P receptors may swing the pendulum in favour of more promising comorbidity treatments. Cer: ceramide, Sph: sphingosine, SphK1: sphingosine kinase 1, S1P: sphingosine-1-phosphate.

Obesity is a common risk factor linking type 2 diabetes and cancer and is covered extensively in a recent review [6]. Type 2 diabetes and obesity have been associated independently, and in common, with increased cancer risk [8]. This risk may be attributed to underlying metabolic conditions such as insulin resistance, hyperinsulinaemia, hyperglycaemia, and inflammation, which all influence the development and progression of neoplasia [26]. Treatment of diabetes with glucose-lowering therapies, such as metformin, has been reviewed extensively and, in general, the treatment of diabetic patients with metformin has been shown to lead to a reduced cancer risk and results in a better overall survival [5, 10, 11, 27]. The effects of cancer drugs on coexisting diabetes have been less well studied and in some cases cancer therapies may cause increased risk of diabetes development [27, 28]. A signalling pathway crucial to the onset/progression of cancer and diabetes is the phosphatidylinositol-3-kinase (PI3K)/AKT/mammalian target of rapamycin (mTOR) pathway [29]. Hyperactivation of this pathway is known to result in increased cell proliferation, decreased apoptosis, and cancer [28]. Inhibitors of this pathway are used for cancer therapy but such drugs may result in impaired insulin responses and insulin resistance leading to the development of type 2 diabetes [28]. Cancer chemotherapy with drugs such as 5-fluorouracil, androgen-deprivation therapy, and carboplatin has been reported to be associated with drug-induced diabetes or the worsening of preexisting diabetes and is reviewed in [6]. More recently, manipulation of the sphingosine kinase/sphingosine-1-phosphate (SphK/S1P) signalling pathway using generic and specific inhibitors has been investigated as a potential cancer therapy [13–15, 30–34]. However, there is a fine balance between swinging the ceramide-SphK/S1P pendulum in favour of cancer prevention/treatment and the onset of diabetes (Figure 1). This conundrum is discussed in more detail below.

## 4. Sphingosine Kinase 

There are two major isoforms of SphK (SphK1 and SphK2) with diverse and compensatory actions [35]. SphK mediates the balance between the proapoptotic effects of ceramide and sphingosine substrates and the antiapoptotic effects of sphingosine-1-phosphate (S1P), a phosphorylation balance system more aptly named “the sphingolipid rheostat” [18, 36, 37]. SphK phosphorylates sphingosine to produce sphingosine-1-phosphate (S1P) and modulates autocrine (intracellular) and paracrine (extracellular) functions. S1P binds mainly to five specific G-protein-coupled receptors (GPCRs), S1P_1−5_ [38]. One or more of the five S1P receptor subtypes are found on the surface of most cells [38]. S1P activation and function is cell type and S1P receptor type specific. In skeletal muscle cells, S1P has been shown to increase glucose uptake through the transactivation of the insulin receptor [39] whereas in epithelial cells S1P inhibits AKT activity and interrupts insulin signalling and cell proliferation through the S1P_2_ receptor subtype [40]. The SphK1 isoform has two major subtypes, SphK1a and SphK1b, and emerging evidence indicates that SphK1a and SphK1b have common and differing interacting partners [41] and, through such interactions, each subtype is able to influence diverse downstream signalling pathways [42]. Tipping the balance in favour of ceramide accumulation has been shown to cause insulin resistance whereas SphK1 prevents ceramide accumulation by promoting its metabolism to S1P and augmenting insulin action [16, 43, 44]. In contrast, overexpression of SphK1 is associated with increased cancer risk [7, 12]. As mentioned previously, inhibitors of SphK1 are currently being explored for cancer treatment; however, with the high probability of comorbidity of cancer and diabetes [5, 6], the possibility of cancer treatments such as SphK1 inhibitors promoting insulin resistance may have dire consequences for cancer survivors.

## 5. SphK and S1P Inhibitors and Diabetes/Obesity Complications 

The drive towards the use of SphK/S1P pharmaceutical inhibitors for cancer treatment has key significance for diabetic patients. The “sphingolipid rheostat” is implicated in controlling the balance between cell proliferation and apoptosis. As such, activation of S1P has been shown to be critical in protecting pancreatic *β*-cells (the cells that produce, store, and release insulin) from apoptosis and preventing the development of diabetes in obese mice [43]. Abnormal islet function is central to the development of type 1 and type 2 diabetes [45]; therefore the danger of SphK/S1P inhibitors for cancer therapy is that they may increase the risk of diabetes development. In support of S1P activation in diabetic control, S1P has been shown to be important for insulin synthesis and secretion in a rat insulinoma cell line [46], muscle insulin resistance [16], and adiponectin action (increased sensitivity, decreased inflammation, and prosurvival) [47, 48]. The diabetic mouse model, KK/Ay, demonstrates a morbidly obese phenotype with metabolic abnormalities that are common in diabetic patients [49]. Overexpression of SphK1 in KK/Ay diabetic mice has been shown to significantly reduce blood glucose levels and improve the overall health of the animals whilst having no effect on normal animals [17].

There is a strong risk of cardiovascular diseases and heart failure in diabetic patients [50–55]. Several studies and reviews have emphasised the importance of SphK1/S1P in cardioprotection [17, 56–58]. A typical feature of the phenotype of animal models of diabetes is an increased accumulation of glycogen in the myocardium which leads to cardiomyopathy [59]. Such glycogen accumulation, which is typical of KK/Ay diabetic mice, was absent after adenoviral mediated (Ad-SphK1) overexpression of SphK1, potentially improving the function of the heart [17]. Moreover, impairment of liver and kidney function associated with the diabetic phenotype was also reversed in the Ad-SphK1 diabetic mice [17].

Atherosclerosis, the hardening of the arteries eventually leading to heart attacks and peripheral vascular disease, is accelerated in type 1 and type 2 diabetic patients [60–62]. Interactions between monocytes and endothelial cells are critical early events in the development of atherosclerosis [63]. In the nonobese diabetic mouse model (NOD/LtJ), a mouse model of spontaneous type 1 diabetes development (autoimmune destruction of the pancreatic islet cells), S1P minimises the monocyte/endothelial interaction that occurs in elevated glucose environments [64, 65].

Silent myocardial ischaemia is frequently presented in diabetic patients and this is reviewed in [66]. Activation of SphK1 has been shown to protect isolated mouse hearts against ischaemia/reperfusion injury [67], to have a cardioprotective effect of ischaemic preconditioning in mice and ischaemia/reperfusion injury [67–69] and to play a role in recovery of haemodynamic function after ischaemic injury [69]. In addition, SphK1 is important in the maintenance of blood vessel integrity and mice depleted of SphK1 have increased vascular leakiness [70]. Wound healing is also problematic in diabetic patients; however, SphK1/S1P activation has recently shown promise in the improvement of the wound healing process in diabetic rats [71].

Prevention of diabetes and improved pancreatic islet transplantation outcomes through pharmacological manipulation of the sphingolipid rheostat in favour of SphK1 has been shown to (i) promote insulin release, (ii) promote establishment and maintenance of intraislet vasculature, (iii) improve glucose sensing, and (iv) play a role in the prevention/treatment of the immune-mediated attack [45]. SphK1/S1P also plays a prosurvival role in primary hepatocytes and protects against liver injury [72].

On the other hand, S1P activation is not all positive for diabetic patients. S1P has been shown to be significantly increased in the blood of obese humans and mice and elevated S1P levels in humans have been correlated with metabolic dysfunction, cardiovascular problems, high body mass index (BMI), and large waist circumference, all factors associated with obesity [73]. Complications associated with obesity are also linked to cancer risk [10]. Wang and colleagues demonstrated that SphK1 overexpression was associated with adipose proinflammatory responses and insulin resistance in diet-induced obese mice and obese diabetic humans [74]. In agreement with these findings Tous and colleagues demonstrated that activation of SphK1 in adipocytes (fat cells) triggered a cytokine inflammatory response whereas suppression of SphK1 activation lowered the expression of proinflammatory cytokines in adipose tissue of Zucker diabetic fatty rats [75]. In these experimental scenarios, inhibition of SphK1 was suggested as a therapeutic tool for the prevention and treatment of inflammation associated with obesity and type 2 diabetes [75]. Although there are several studies and reviews emphasising the importance of SphK1/S1P in cardioprotection (as mentioned above), elevated SphK1/S1P levels have also been associated with the negative effects of cardiovascular diseases linked to diabetes. For example, in one study SphK1 inhibition ameliorated angiotensin II-induced acute hypertension [76] and in another study deregulation of specific S1Ps played a role in cardiac microvascular dysfunction [77]. A growing list of adverse diabetic complications is believed to be involved with high levels of SphK1/S1P expression including neuropathy [36, 78, 79], retinopathy [80–84], nephropathy [85], and cancer [5, 6, 8]. The complexities of insulin resistance, with reference to the onset of diabetes and the modulation of S1P signalling, are discussed comprehensively in recent articles by Fayyaz and colleagues [86, 87]. In summary, the major apparent hurdle is that therapies targeting the SphK/S1P rheostat in cancer patients (for cancer therapy) may prove to be a double-edged sword where predisposing conditions such as obesity and diabetes are also presented. In addition, complications associated with the use of SphK1/S1P inhibitors may be that cancer patients are more susceptible to diabetes development. The multifaceted nature of SphK complicates the generation of SphK/S1P inhibitors as therapies for cancer.

## 6. SphK and S1P Inhibitors: Obesity/Diabetes/Cancer Conundrum

The development of treatment regimes to avoid complications arising from the presence of combined disease states, such as cancer and diabetes, is a major challenge: in this case, to balance cancer cell apoptosis and reduce disease complications whilst protecting pancreatic *β*-cell proliferation, it is becoming increasingly apparent that balancing the sphingosine rheostat is crucial in the development of many types of cancer and also diabetes; however, the opposing effects of SphK/S1P inhibitors on diabetes and cancer are a conundrum. It is unknown whether S1P activation influences both type 1 and type 2 diabetes outcomes such as mechanism of *β*-cell death or insulin resistance in skeletal muscle. Furthermore, obese cancer patients could be at heightened risk of diabetes if treated with SphK/S1P inhibitors and this concept needs to be considered in future research in SphK/S1P inhibitor design and treatment. S1P agonists and functional antagonists (S1P receptor modulators) are in development to target specific S1P receptor subtypes to maximise therapeutic efficacy [88, 89]. FTY720 (fingolimod) is a first generation S1P modulator under consideration for the treatment of cancer and diabetes, however not necessarily for comorbidity therapy. FTY720 is a S1P analogue that mimics S1P as an agonist of all the S1P receptors except S1P_2_ [90–92]. Despite this, it also acts as a functional S1P_1_ receptor antagonist, reviewed in [93]. The fact that FTY720 does not bind to S1P_2_ has created much interest for diabetes/cancer therapy advocates. There are mixed results reported to date with the use of FTY720 for cancer treatment. Recent advances have shown the use of FTY720 and its derivatives to be promising potential therapies for cancers such as intestinal and colorectal cancer [94–97], leukaemia [95, 98, 99], ovarian cancer [100], triple-negative breast cancers [101], and increased sensitivity to radiation of breast cancer cells [102]. Moreover, FTY720 inhibits melanoma growth and invasion in 3D culture* in vitro* (NKH, unpublished results). On the other hand, FTY720 decreased sensitivity of breast cancer cells overexpressing the oncogene pp32r1 [103] and HER2 targeted therapy with lapatinib [104] potentially compromising the efficacy of FTY720 in some breast cancer clinical cotreatment regimes.

**Figure 2:**
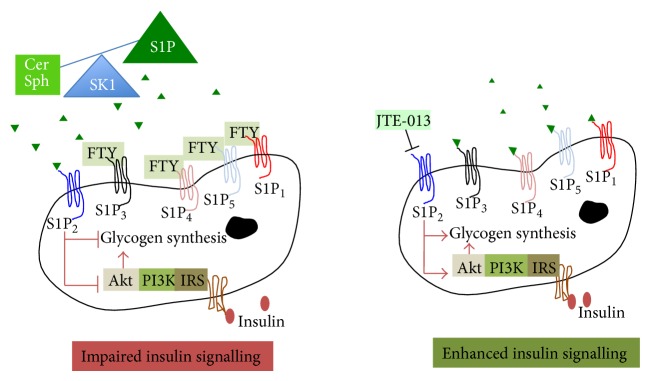
The S1P_2_ receptor modulates hepatic insulin signalling. FTY720 binds to S1P_1,3−5 _receptors and does not impact the normal signalling functions of S1P_2_. S1P_2_ has been associated with impaired insulin signalling [86, 109]. FTY720 is a S1P_1,3−5 _agonist but also acts as a functional antagonist of S1P_1_ [109]. FTY720 does not bind to S1P_2_ and therefore does not affect S1P_2_ function. In contrast, JTE-013 inhibits S1P_2_.

SphK1/S1P inhibitors as therapies for diabetes are also problematic. The effect of FTY720 in various animal models of type 1 diabetes is summarised by Jessup and colleagues [45]. The efficacy of FTY720 ranges from complete prevention of diabetes, short-term prevention, and—depending on the disease stage and time point of drug administration—diminished efficacy from 20–100% [45]. In recent studies, FTY720 has been shown to inhibit the development of obesity in high fat fed mice, by modulation of adipogenesis and lipolysis [105], and to attenuate the accumulation of ceramide in muscles, associated with a high fat diet, resulting in improved whole body glucose homeostasis [106] and amelioration of prediabetic type 2 disposition. Previous reports also provided promising results with complete reversal of diabetes (6/11 mice) in obese mice with continuous administration of FTY720 [107]. In addition, the recent study by Moon and colleagues demonstrated that FTY720 increased *β*-cell survival and restored *β*-cell function with improved glucose tolerance in a diabetic* (db/db)* mouse model [108]. Not all groups have found FTY720 beneficial in the prevention or cure of diabetes [86, 109]. Fayyaz and colleagues demonstrated FTY720 was unable to modulate S1P mediated insulin signalling in human and rat hepatocytes [86]. As mentioned, FTY720 does not bind the S1P_2_ receptor. The importance of the S1P_2_ receptor in insulin resistance was demonstrated by blocking the receptor using a specific antagonist (JTE-013), thereby increasing hepatic insulin signalling [86, 109]. Hence specific S1P_2_ receptor antagonists such as JTE-013 have been suggested as targets for diabetes treatments (Figure 2).

**Figure 3:**
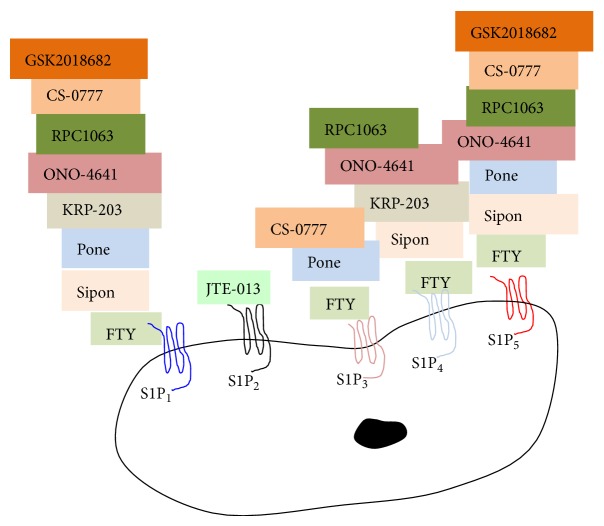
Balancing the SphK1/S1P rheostat for diabetes and cancer comorbidity treatments. Second generation S1P receptor modulators are currently being developed to target individual and multiple S1P receptors. Each of the receptor modulators binds to individual or multiple receptors to block or activate the S1P receptor. Siponimod is a S1P_1,4,5_ modulator; ponesimod is an agonist for S1P_1,3,4_; KRP-203 is an agonist for S1P_1,4_; ONO-4641 is an agonist for S1P_1,4,5_; RPC1063 is an agonist for S1P_1,4,5_; Cs-0777 is an agonist for S1P_1,3,5_; GSK2018682 is an agonist for S1P_1,5_. FTY720 and JTE-013 are described in Figure 2. These novel S1P receptors and downstream signalling pathways and functions are reviewed in [88, 93]. Sipon: siponimod; pone: ponesimod; FTY: FTY720.

The controversial function of current S1P agonists and functional antagonists has been associated with binding of differing S1P receptor transmembrane expression, such as demonstrated for FTY720. As discussed above, SphK1/S1P inhibitors can have positive and negative impact for diabetic patients depending on the patient's specific condition. Current second generation S1P receptor agonists hold much promise for comorbidity cancer/diabetes treatments and are reviewed in [88, 89]. A comparison of fingolimod (FTY720) and the most advanced next generation S1P modulators (siponimod, ponesimod, KRP-203, ONO-4641, RPC1063, CS-0777, and GSK2018682), each modulator targeting common and different S1P receptors, are illustrated in Figure 3 [88, 93]. Comparative selectivity of S1P modulator activation of specific S1P receptors is shown in Table 1. Knowledge of specific S1P receptor function provides some insight into how S1P receptor modulators may be targeted for comorbidity treatments.

## 7. The SphK1/S1P Rheostat Therapeutic Challenge

Targeting the sphingolipid rheostat for diabetes and cancer therapy holds great promise; however, the treatment for comorbidity will be the greatest hurdle to overcome. As portrayed in Figure 1, the challenge will be to balance cancer cell apoptosis on the one hand and promote *β*-cell survival for insulin production on the other hand; it is a swinging pendulum (Figure 1). A greater understanding of the actions of SphK1/S1P in the context of diabetes, especially the onset of type 2 diabetes and cancer, is required if we are to switch the sphingolipid rheostat in the treatment of diabetes and cancer comorbidity from a problem to an advantage.

## Figures and Tables

**Table 1:** Comparative selectivity of the S1P modulators (adapted from [88]).

	S1P_1_	S1P_2_	S1P_3_	S1P_4_	S1P_5_
FTY720	++++	−	+	++	+++
CS-0777	+++	−	+	−	++
Ponesimod	+++	−	+	−	++
RPC0163	++++	+/−	+/−	+/−	++
ONO-4641	++	−	−	+	++
Siponimod	++	−	−	+	++
GSK2018682	++	−	−	−	+
KRP-203	+++	−	−	++	−
JTE-013	−	+++	−	−	−

+ indicates comparative selectivity of S1P modulators binding to individual receptors.

## References

[1] Danaei G., Finucane M. M., Lu Y. (2011). National, regional, and global trends in fasting plasma glucose and diabetes prevalence since 1980: systematic analysis of health examination surveys and epidemiological studies with 370 country-years and 2·7 million participants. *The Lancet*.

[2] WHO (2011). *Global Status Report on Noncommunicable Diseases 2010*.

[3] Ferlay J., Soerjomataram I., Ervik M. (2012). *GLOBOCAN 2012 v1.0*.

[4] Bray F., Ren J.-S., Masuyer E., Ferlay J. (2013). Global estimates of cancer prevalence for 27 sites in the adult population in 2008. *International Journal of Cancer*.

[5] Sen S., He Y., Koya D., Kanasaki K. (2014). Cancer biology in diabetes. *Journal of Diabetes Investigation*.

[6] Garg S. K., Maurer H., Reed K., Selagamsetty R. (2014). Diabetes and cancer: two diseases with obesity as a common risk factor. *Diabetes, Obesity and Metabolism*.

[7] Khandekar M. J., Cohen P., Spiegelman B. M. (2011). Molecular mechanisms of cancer development in obesity. *Nature Reviews Cancer*.

[8] Giovannucci E., Harlan D. M., Archer M. C. (2010). Diabetes and cancer: a consensus report. *Diabetes Care*.

[9] Barone B. B., Yeh H.-C., Snyder C. F. (2008). Long-term all-cause mortality in cancer patients with preexisting diabetes mellitus: a systematic review and meta-analysis. *The Journal of the American Medical Association*.

[10] Noto H., Goto A., Tsujimoto T., Osame K., Noda M. (2013). Latest insights into the risk of cancer in diabetes. *Journal of Diabetes Investigation*.

[11] Jalving M., Gietema J. A., Lefrandt J. D. (2010). Metformin: taking away the candy for cancer?. *European Journal of Cancer*.

[12] Maceyka M., Harikumar K. B., Milstien S., Spiegel S. (2012). Sphingosine-1-phosphate signaling and its role in disease. *Trends in Cell Biology*.

[13] Truman J.-P., García-Barros M., Obeid L. M., Hannun Y. A. (2014). Evolving concepts in cancer therapy through targeting sphingolipid metabolism. *Biochimica et Biophysica Acta*.

[14] Heffernan-Stroud L. A., Obeid L. M. (2013). Sphingosine kinase 1 in cancer. *Advances in Cancer Research*.

[15] Plano D., Amin S., Sharma A. K. (2014). Importance of sphingosine kinase (SphK) as a target in developing cancer therapeutics and recent developments in the synthesis of novel SphK inhibitors. *Journal of Medicinal Chemistry*.

[16] Bruce C. R., Risis S., Babb J. R. (2012). Overexpression of sphingosine kinase 1 prevents ceramide accumulation and ameliorates muscle insulin resistance in high-fat diet-fed mice. *Diabetes*.

[17] Ma M. M., Chen J. L., Wang G. G. (2007). Sphingosine kinase 1 participates in insulin signalling and regulates glucose metabolism and homeostasis in KK/Ay diabetic mice. *Diabetologia*.

[18] Hannun Y. A., Obeid L. M. (2008). Principles of bioactive lipid signalling: lessons from sphingolipids. *Nature Reviews Molecular Cell Biology*.

[19] Olivera A., Spiegel S. (1993). Sphingosine-1-phosphate as second messenger in cell proliferation induced by PDGF and FCS mitogens. *Nature*.

[20] van Brocklyn J. R., Lee M.-J., Menzeleev R. (1998). Dual actions of sphingosine-1-phosphate: extracellular through the G_i_- coupled receptor Edg-1 and intracellular to regulate proliferation and survival. *The Journal of Cell Biology*.

[21] (2003). Report of the expert committee on the diagnosis and classification of diabetes mellitus. *Diabetes Care*.

[22] Cnop M., Welsh N., Jonas J.-C., Jörns A., Lenzen S., Eizirik D. L. (2005). Mechanisms of pancreatic *β*-cell death in type 1 and type 2 diabetes: many differences, few similarities. *Diabetes*.

[23] Shao S., Yang Y., Yuan G., Zhang M., Yu X. (2013). Signaling molecules involved in lipid-induced pancreatic beta-cell dysfunction. *DNA and Cell Biology*.

[24] Newsholme P., Keane D., Welters H. J., Morgan N. G. (2007). Life and death decisions of the pancreatic *β*-cell: the role of fatty acids. *Clinical Science*.

[25] Phielix E., Mensink M. (2008). Type 2 diabetes mellitus and skeletal muscle metabolic function. *Physiology and Behavior*.

[26] Johnson J. A., Pollak M. (2010). Insulin, glucose and the increased risk of cancer in patients with type 2 diabetes. *Diabetologia*.

[27] Li C., Kong D. (2014). Cancer risks from diabetes therapies: evaluating the evidence. *Pharmacology & Therapeutics*.

[28] Braccini L., Ciraolo E., Martini M. (2012). PI3K keeps the balance between metabolism and cancer. *Advances in Biological Regulation*.

[29] Zoncu R., Efeyan A., Sabatini D. M. (2011). mTOR: From growth signal integration to cancer, diabetes and ageing. *Nature Reviews Molecular Cell Biology*.

[30] Orr Gandy K. A., Obeid L. M. (2013). Targeting the sphingosine kinase/sphingosine 1-phosphate pathway in disease: review of sphingosine kinase inhibitors. *Biochimica et Biophysica Acta: Molecular and Cell Biology of Lipids*.

[31] Pyne N. J., Pyne S. (2010). Sphingosine 1-phosphate and cancer. *Nature Reviews Cancer*.

[32] Pyne N. J., Tonelli F., Lim K. G. (2012). Targeting sphingosine kinase 1 in cancer. *Biochemical Society Transactions*.

[33] Hannun Y. A., Loomis C. R., Merrill A. H., Bell R. M. (1986). Sphingosine inhibition of protein kinase C activity and of phorbol dibutyrate binding in vitro and in human platelets. *The Journal of Biological Chemistry*.

[34] Milstien S., Spiegel S. (2006). Targeting sphingosine-1-phosphate: a novel avenue for cancer therapeutics. *Cancer Cell*.

[35] Alemany R., van Koppen C. J., Danneberg K., Ter Braak M., Zu Heringdorf D. M. (2007). Regulation and functional roles of sphingosine kinases. *Naunyn-Schmiedeberg's Archives of Pharmacology*.

[36] Abuhusain H. J., Matin A., Qiao Q. (2013). A metabolic shift favoring sphingosine 1-phosphate at the expense of ceramide controls glioblastoma angiogenesis. *The Journal of Biological Chemistry*.

[37] Hait N. C., Oskeritzian C. A., Paugh S. W., Milstien S., Spiegel S. (2006). Sphingosine kinases, sphingosine 1-phosphate, apoptosis and diseases. *Biochimica et Biophysica Acta*.

[38] Mendelson K., Evans T., Hla T. (2014). Sphingosine 1-phosphate signalling. *Development*.

[39] Rapizzi E., Taddei M. L., Fiaschi T., Donati C., Bruni P., Chiarugi P. (2009). Sphingosine 1-phosphate increases glucose uptake through trans-activation of insulin receptor. *Cellular and Molecular Life Sciences*.

[40] Schüppel M., Kürschner U., Kleuser U., Schäfer-Korting M., Kleuser B. (2008). Sphingosine 1-phosphate restrains insulin-mediated keratinocyte proliferation via inhibition of Akt through the S1P2 receptor subtype. *The Journal of Investigative Dermatology*.

[41] Yagoub D., Wilkins M. R., Lay A. J. (2014). Sphingosine kinase 1 isoform-specific interactions in breast cancer. *Molecular Endocrinology*.

[42] Lim K. G., Tonelli F., Berdyshev E. (2012). Inhibition kinetics and regulation of sphingosine kinase 1 expression in prostate cancer cells: functional differences between sphingosine kinase 1a and 1b. *The International Journal of Biochemistry & Cell Biology*.

[43] Qi Y., Chen J., Lay A., Don A., Vadas M., Xia P. (2013). Loss of sphingosine kinase 1 predisposes to the onset of diabetes via promoting pancreatic *β*-cell death in diet-induced obese mice. *FASEB Journal*.

[44] Holland W. L., Brozinick J. T., Wang L.-P. (2007). Inhibition of ceramide synthesis ameliorates glucocorticoid-, saturated-fat-, and obesity-induced insulin resistance. *Cell Metabolism*.

[45] Jessup C. F., Bonder C. S., Pitson S. M., Coates P. T. H. (2011). The sphingolipid rheostat: a potential target for improving pancreatic islet survival and function. *Endocrine, Metabolic and Immune Disorders: Drug Targets*.

[46] Hasan N. M., Longacre M. J., Stoker S. W. (2012). Sphingosine kinase 1 knockdown reduces insulin synthesis and secretion in a rat insulinoma cell line. *Archives of Biochemistry and Biophysics*.

[47] Holland W. L., Miller R. A., Wang Z. V. (2011). Receptor-mediated activation of ceramidase activity initiates the pleiotropic actions of adiponectin. *Nature Medicine*.

[48] Tao C., Sifuentes A., Holland W. L. (2014). Regulation of glucose and lipid homeostasis by adiponectin: effects on hepatocytes, pancreatic *β* cells and adipocytes. *Best Practice & Research: Clinical Endocrinology and Metabolism*.

[49] Iwatsuka H., Shino A., Suzuoki Z. (1970). General survey of diabetic features of yellow KK mice. *Endocrinologia Japonica*.

[50] Schnell O., Cappuccio F., Genovese S., Standl E., Valensi P., Ceriello A. (2013). Type 1 diabetes and cardiovascular disease. *Cardiovascular Diabetology*.

[51] Paneni F., Beckman J. A., Creager M. A., Cosentino F. (2013). Diabetes and vascular disease: pathophysiology, clinical consequences, and medical therapy: part I. *European Heart Journal*.

[52] Grundy S. M., Benjamin I. J., Burke G. L. (1999). Diabetes and cardiovascular disease: a statement for healthcare professionals from the american heart association. *Circulation*.

[53] Fioretto P., Dodson P. M., Ziegler D., Rosenson R. S. (2010). Residual microvascular risk in diabetes: unmet needs and future directions. *Nature Reviews Endocrinology*.

[54] Paneni F., Costantino S., Cosentino F. (2014). Insulin resistance, diabetes, and cardiovascular risk. *Current Atherosclerosis Reports*.

[55] Li H., Horke S., Forstermann U. (2014). Vascular oxidative stress, nitric oxide and atherosclerosis. *Atherosclerosis*.

[56] Keul P., Sattler K., Levkau B. (2007). HDL and its sphingosine-1-phosphate content in cardioprotection. *Heart Failure Reviews*.

[57] Karliner J. S. (2009). Sphingosine kinase regulation and cardioprotection. *Cardiovascular Research*.

[58] Karliner J. S. (2013). Sphingosine kinase and sphingosine 1-phosphate in the heart: a decade of progress. *Biochimica et Biophysica Acta: Molecular and Cell Biology of Lipids*.

[59] Penpargkul S., Schaible T., Yipintsoi T., Scheuer J. (1980). The effect of diabetes on performance and metabolism of rat hearts. *Circulation Research*.

[60] Dahl-Jørgensen K., Larsen J. R., Hanssen K. F. (2005). Atherosclerosis in childhood and adolescent type 1 diabetes: early disease, early treatment?. *Diabetologia*.

[61] Fisher M. (2004). Diabetes and atherogenesis. *Heart*.

[62] Donnelly R., Davis K. R. (2000). Type 2 diabetes and atherosclerosis. *Diabetes, Obesity and Metabolism*.

[63] Mestas J., Ley K. (2008). Monocyte-endothelial cell interactions in the development of atherosclerosis. *Trends in Cardiovascular Medicine*.

[64] Whetzel A. M., Bolick D. T., Hedrick C. C. (2009). Sphingosine-1-phosphate inhibits high glucose-mediated ERK1/2 action in endothelium through induction of MAP kinase phosphatase-3. *American Journal of Physiology: Cell Physiology*.

[65] Whetzel A. M., Bolick D. T., Srinivasan S. (2006). Sphingosine-1 phosphate prevents monocyte/endothelial interactions in type 1 diabetic NOD mice through activation of the S1P1 receptor. *Circulation Research*.

[66] Khafaji H. A., Suwaidi J. M. (2014). A typical presentation of acute and chronic coronary artery disease in diabetics. *World Journal of Cardiology*.

[67] Jin Z.-Q., Karliner J. S., Vessey D. A. (2008). Ischaemic postconditioning protects isolated mouse hearts against ischaemia/reperfusion injury via sphingosine kinase isoform-1 activation. *Cardiovascular Research*.

[68] Vessey D. A., Kelley M., Li L., Huang Y. (2009). Sphingosine protects aging hearts from ischemia/reperfusion injury: superiority to sphingosine 1-phosphate and ischemic pre- and post-conditioning. *Oxidative Medicine and Cellular Longevity*.

[69] Vessey D. A., Kelley M., Li L. (2006). Role of sphingosine kinase activity in protection of heart against ischemia reperfusion injury. *Medical Science Monitor*.

[70] Bonder C. S., Sun W. Y., Matthews T. (2009). Sphingosine kinase regulates the rate of endothelial progenitor cell differentiation. *Blood*.

[71] Yu H., Yuan L., Xu M., Zhang Z., Duan H. (2014). Sphingosine kinase 1 improves cutaneous wound healing in diabetic rats. *Injury*.

[72] Osawa Y., Uchinami H., Bielawski J., Schwabe R. F., Hannun Y. A., Brenner D. A. (2005). Roles for C16-ceramide and sphingosine 1-phosphate in regulating hepatocyte apoptosis in response to tumor necrosis factor-*α*. *The Journal of Biological Chemistry*.

[73] Kowalski G. M., Carey A. L., Selathurai A., Kingwell B. A., Bruce C. R. (2013). Plasma sphingosine-1-phosphate is elevated in obesity. *PLoS ONE*.

[74] Wang J., Badeanlou L., Bielawski J., Ciaraldi T. P., Samad F. (2014). Sphingosine kinase 1 regulates adipose proinflammatory responses and insulin resistance. *American Journal of Physiology: Endocrinology and Metabolism*.

[75] Tous M., Ferrer-Lorente R., Badimon L. (2014). Selective inhibition of sphingosine kinase-1 protects adipose tissue against LPS-induced inflammatory response in Zucker diabetic fatty rats. *The American Journal of Physiology, Endocrinology and Metabolism*.

[76] Furuya H., Wada M., Shimizu Y. (2013). Effect of sphingosine kinase 1 inhibition on blood pressure. *The FASEB Journal*.

[77] Yin Z., Fan L., Wei L. (2012). FTY720 protects cardiac microvessels of diabetes: a critical role of S1P1/3 in diabetic heart disease. *PLoS ONE*.

[78] Janes K., Little J. W., Li C. (2014). The development and maintenance of paclitaxel-induced neuropathic pain require activation of the sphingosine 1-phosphate receptor subtype 1. *The Journal of Biological Chemistry*.

[79] Guan H., Song L., Cai J. (2011). Sphingosine kinase 1 regulates the Akt/FOXO3a/Bim pathway and contributes to apoptosis resistance in glioma cells. *PLoS ONE*.

[80] Xie B., Shen J., Dong A., Rashid A., Stoller G., Campochiaro P. A. (2009). Blockade of sphingosine-1-phosphate reduces macrophage influx and retinal and choroidal neovascularization. *Journal of Cellular Physiology*.

[81] Maines L. W., French K. J., Wolpert E. B., Antonetti D. A., Smith C. D. (2006). Pharmacologic manipulation of sphingosine kinase in retinal endothelial cells: implications for angiogenic ocular diseases. *Investigative Ophthalmology and Visual Science*.

[82] Sabbadini R. A. (2011). Sphingosine-1-phosphate antibodies as potential agents in the treatment of cancer and age-related macular degeneration. *British Journal of Pharmacology*.

[83] Visentin B., Vekich J. A., Sibbald B. J. (2006). Validation of an anti-sphingosine-1-phosphate antibody as a potential therapeutic in reducing growth, invasion, and angiogenesis in multiple tumor lineages. *Cancer Cell*.

[84] Mizugishi K., Yamashita T., Olivera A., Miller G. F., Spiegel S., Proia R. L. (2005). Essential role for sphingosine kinases in neural and vascular development. *Molecular and Cellular Biology*.

[85] Lan T., Liu W., Xie X. (2011). Sphingosine kinase-1 pathway mediates high glucose-induced fibronectin expression in glomerular mesangial cells. *Molecular Endocrinology*.

[86] Fayyaz S., Henkel J., Japtok L. (2014). Involvement of sphingosine 1-phosphate in palmitate-induced insulin resistance of hepatocytes via the S1P2 receptor subtype. *Diabetologia*.

[87] Fayyaz S., Japtok L., Kleuser B. (2014). Divergent role of sphingosine 1-phosphate on insulin resistance. *Cellular Physiology and Biochemistry*.

[88] Bigaud M., Guerini D., Billich A., Bassilana F., Brinkmann V. (2014). Second generation S1P pathway modulators: research strategies and clinical developments. *Biochimica et Biophysica Acta: Molecular and Cell Biology of Lipids*.

[89] Huwiler A., Pfeilschifter J. (2008). New players on the center stage: sphingosine 1-phosphate and its receptors as drug targets. *Biochemical Pharmacology*.

[90] Bandhuvula P., Tam Y. Y., Oskouian B., Saba J. D. (2005). The immune modulator FTY720 inhibits sphingosine-1-phosphate lyase activity. *The Journal of Biological Chemistry*.

[91] Kaneider N. C., Lindner J., Feistritzer C. (2004). The immune modulator FTY720 targets sphingosine-kinase-dependent migration of human monocytes in response to amyloid beta-protein and its precursor. *The FASEB Journal*.

[92] Brinkmann V., Davis M. D., Heise C. E. (2002). The immune modulator FTY720 targets sphingosine 1-phosphate receptors. *Journal of Biological Chemistry*.

[93] Blaho V. A., Hla T. (2014). An update on the biology of sphingosine 1-phosphate receptors. *The Journal of Lipid Research*.

[94] Cristobal I., Manso R., Rincon R. (2014). PP2A inhibition is a common event in colorectal cancer and its restoration using FTY720 shows promising therapeutic potential. *Molecular Cancer Therapeutics*.

[95] Neviani P., Santhanam R., Oaks J. J. (2007). FTY720, a new alternative for treating blast crisis chronic myelogenous leukemia and Philadelphia chromosome-positive acute lymphocytic leukemia. *The Journal of Clinical Investigation*.

[96] Nagahashi M., Hait N. C., Maceyka M. (2014). Sphingosine-1-phosphate in chronic intestinal inflammation and cancer. *Advances in Biological Regulation*.

[97] Liang J., Nagahashi M., Kim E. Y. (2013). Sphingosine-1-phosphate links persistent STAT3 activation, chronic intestinal inflammation, and development of colitis-associated cancer. *Cancer Cell*.

[98] Liu Q., Zhao X., Frissora F. (2008). FTY720 demonstrates promising preclinical activity for chronic lymphocytic leukemia and lymphoblastic leukemia/lymphoma. *Blood*.

[99] Mani R., Mao Y., Frissora F. W. (2014). Tumor antigen ROR1 targeted drug delivery mediated selective leukemic but not normal B cell cytotoxicity in chronic lymphocytic leukemia. *Leukemia*.

[100] Zhang N., Qi Y., Wadham C. (2010). FTY720 induces necrotic cell death and autophagy in ovarian cancer cells: a protective role of autophagy. *Autophagy*.

[101] Baldacchino S., Saliba C., Petroni V. (2014). Deregulation of the phosphatase, PP2A is a common event in breast cancer, predicting sensitivity to FTY720. *EPMA Journal*.

[102] Marvaso G., Barone A., Amodio N. (2014). Sphingosine analog fingolimod (FTY720) increases radiation sensitivity of human breast cancer cells in vitro. *Cancer Biology & Therapy*.

[103] Buddaseth S., Göttmann W., Blasczyk R., Huyton T. (2014). Overexpression of the pp32r1 (ANP32C) oncogene or its functional mutant pp32r1Y140H confers enhanced resistance to FTY720 (Finguimod). *Cancer Biology and Therapy*.

[104] McDermott M. S., Browne B. C., Conlon N. T. (2014). PP2A inhibition overcomes acquired resistance to HER2 targeted therapy. *Molecular Cancer*.

[105] Park S.-Y., Moon M.-H., Jeong J.-K. (2012). Antiobesity activity of a sphingosine 1-phosphate analogue FTY720 observed in adipocytes and obese mouse model. *Experimental and Molecular Medicine*.

[106] Bruce C. R., Risis S., Babb J. R. (2013). The sphingosine-1-phosphate analog FTY720 reduces muscle ceramide content and improves glucose tolerance in high fat-fed male mice. *Endocrinology*.

[107] Maki T., Gottschalk R., Ogawa N., Monaco A. P. (2005). Prevention and cure of autoimmune diabetes in nonobese diabetic mice by continuous administration of FTY720. *Transplantation*.

[108] Moon H., Chon J., Joo J. (2013). FTY720 preserved islet *β*-cell mass by inhibiting apoptosis and increasing survival of *β*-cells in db/db mice. *Diabetes/Metabolism Research and Reviews*.

[109] Imasawa T., Koike K., Ishii I., Chun J., Yatomi Y. (2010). Blockade of sphingosine 1-phosphate receptor 2 signaling attenuates streptozotocin-induced apoptosis of pancreatic *β*-cells. *Biochemical and Biophysical Research Communications*.

